# Risk Assessment of Varicose Veins Among Teachers in Al-Ahsa, Saudi Arabia

**DOI:** 10.7759/cureus.26125

**Published:** 2022-06-20

**Authors:** Zaki Busbaih, Ali A Almohammed Saleh, Ahmed H Alsulaiman, Mohammed A Almuhanna, Shatha H AlKhawajah, Shdn B Alsuwayie

**Affiliations:** 1 General Surgery, Prince Saud Bin Jalawi Hospital, Al-Ahsa, SAU; 2 Medical School, King Faisal University, Al-Ahsa, SAU

**Keywords:** varicose veins, vascular lesions, risk assessment, prolonged standing, teachers

## Abstract

Background

Varicose veins (VV) are abnormally swollen, tortuous, and prominent veins caused by insufficient venous valves leading to venous congestion and elevated venous pressure. Prolonged standing at work has been proposed to be an important risk factor for varicose veins. Teachers are prone to have varicose veins due to prolonged standing. The aim of this study was to assess the risk of varicose veins among teachers in Al-Asha, Saudi Arabia.

Method

This was a cross-sectional study conducted among teachers of primary, secondary, and high schools in Al-Ahsa, of both genders, between April 2022 and June 2022. The participants were interviewed and examined for the presence of signs and symptoms of the disease. Finally, the collected data were analyzed using Statistical Package for Social Sciences (SPSS Inc., Chicago, IL, version 26.0 for Windows) software.

Result

Out of 399 participating teachers with a mean age of 43.2 ± 12.9 years, 216 (54.1%) were males and the remainder were female. The commonest symptom was pain in the legs, which was exacerbated by work (43.1%). Most of the participants were working for more than 16 years (43.6%) and standing for less than six hours per day (72.7%). The most pointed sign was spider legs-shaped veins (23.8%). However, the least collective sign was paleness in the ulcer area after healing (1.8%). A total of 140 teachers had a family history of varicose veins, 74 of them (18.5%) were diagnosed with varicose veins previously. We summarize our result as female teachers who have a family history of VV have more risk to develop the disease.

Conclusion

The prevalence of varicose veins was high among teachers in Al-Ahsa, Saudi Arabia. According to our study, teachers have a significant chance of developing the condition since their working style contributes to its progression. Further actions need to be made in order to increase awareness and prevent its complications.

## Introduction

Varicose veins (VV) are abnormally swollen, tortuous, and apparent veins caused by insufficient venous valves leading to venous congestion and elevated venous pressure. They are more frequent in the lower extremities due to saphenous vein insufficiency [1]. The incidence of varicose veins increases with old age, overweight, pregnancy, constipation, smoking, positive family history of VV, history of venous thrombosis, and heavy lifting [1-2].

Prolonged standing at work has been proposed to be an important risk factor for varicose veins [3-4]. As a result, we conclude that the type of one's employment and one's body posture are two of the most important risk factors for varicose veins [5]. Teachers, traffic police, bus conductors, salespeople, nurses, and construction workers are among the highest occupations in developing varicose veins due to their requirements of standing for a long time [1]. Prolonged standing may result in an injury to the valves in superficial and deep veins, which leads to an abnormally opposite direction of blood flow from deep to superficial veins [6]. Pain, itching, heavy sensation in the legs, skin discoloration, and prominent veins are among the most prevalent complaints of such people [7]. Thrombophlebitis and bleeding are the most direct effects, whereas indirect problems include skin color changes, lipodermatosclerosis, atrophie blanche, edema, varicose eczema, and venous ulceration [8]. Besides these physical complications, it has a negative psychological and financial impact. It can result in low self-esteem and social isolation, consequently increasing the risk of depression. Furthermore, patients' everyday activities may be disrupted due to the restricted standing time they have or the limited walking distance, which leads to a lowering in their productivity [9-10]. Certain preventative steps may be taken to reduce the risk of varicose veins such as raising the legs above the level of the heart for several minutes a day, walking regularly, maintaining body weight, and wearing a compression stocking [11].

The prevalence of VV varies between 10% and 60% globally, and it is higher in Asia than in the Western world, with Saudi Arabia having a prevalence of 62% [12]. In Saudi Arabia, there were insufficient studies that delineate the association between VV and the nature of occupation or the number of hours sitting or standing at work, as well as lifestyle variables like smoking and exercising [12-14]. So, our aim is to assess the risk of varicose veins among teachers in Al-Ahsa, Saudi Arabia.

## Materials and methods

A cross-sectional study was conducted at 12 schools selected randomly in Al-Ahsa between April 2022 and June 2022. Institutional ethical approval was obtained before conducting the study from King Faisal University. The questionnaire was distributed by the participants face-to-face with leg examination. A total of 399 school teachers were selected for the study after obtaining their consent. The purpose of the study was explained to all the participants. All their queries and concerns were addressed before administering the questionnaire.

The questionnaire is taken from Robin Man Karmacharya’s research [6]. It consists of three parts. The first part consisted of personal and demographic information such as age, gender, weight, and height. The second part consisted of workplace-related information, which included the duration of service and work responsibilities. The third part consisted of information regarding varicose veins, which include the symptoms and examination of the leg findings.

Inclusion criteria

Male and female teachers working in Al-Ahsa, teaching any grade (primary, secondary, and high schools) at any age.

Exclusion criteria

Teachers who have taught for less than four years.

Data analysis

The data were entered into Microsoft Excel and then analyzed using the Statistical Package for the Social Sciences Version (SPSS Inc., Chicago, IL, version 26.0 for Windows). All data were expressed as frequency and percentage. Significance was determined using the t-test and chi-square test. Linear regression analysis was done to calculate the odds ratio with a 95% confidence interval (CI). P < 0.05 was considered statistically significant. After data were extracted, it was revised, coded, and fed to statistical software IBM SPSS version 22. All statistical analysis was done using two-tailed tests. P-value less than 0.05 was statistically significant. Descriptive analysis based on frequency and percent distribution was done for all variables, including teachers’ demographic data, teaching grade, teaching years, standing hours, and body mass index (BMI). Teachers’ medical and family history of varicose veins, as well as a smoking habit, were also tabulated. Clinical symptoms and signs of varicose veins were graphed. Cross tabulation was used to assess the relation and distribution of varicose vein signs and symptoms by standing hours and teaching years and to identify factors that are associated with VV among teachers. Relations were tested using Pearsons' chi-square test and exact probability test for small frequency distributions.

## Results

A total of 399 teachers fulfilling the inclusion criteria completed the study questionnaire. Teachers' ages ranged from 20 to 60 years with a mean age of 43.2 ± 12.9 years old. An exact of 127 (31.8%) were teachers in the primary grade, 149 (37.3%) in the intermediate grade, and 123 (30.8%) in the secondary grade. A total of 216 (54.1%) teachers were males and 183 (45.9%) were females. As for teaching years, 34 (8.5%) taught for four to seven years, 97 (24.3%) taught for 8-11, and 174 (43.6%) taught for 16 years or more. A total of 290 (72.7%) stood for six hours or less daily while 109 (27.3%) stood for seven hours or more. An exact of 206 (51.6%) were overweight and 109 (27.3%) were obese (Table 1).

**Table 1:** Personal data of the participants

Personal data	No	%
Grade		
Primary	127	31.8%
Intermediate	149	37.3%
Secondary	123	30.8%
Age in years		
20-29	11	2.8%
30-39	101	25.3%
40-49	214	53.6%
50+	73	18.3%
Gender		
Male	216	54.1%
Female	183	45.9%
Teaching years		
4-7	34	8.5%
8-11	97	24.3%
12-15	94	23.6%
16+	174	43.6%
Standing hours / day		
< 6 hours	290	72.7%
> 7 hours	109	27.3%
Body mass index		
Normal	84	21.1%
Overweight	206	51.6%
Obese	109	27.3%

Table 2 represents the medical and family history of varicose veins among teachers, Al-Ahsa, Saudi Arabia. A total of 140 (35.1%) teachers had a family history of varicose veins, 74 (18.5%) were diagnosed with varicose veins previously and 93 of teachers (23.3%) were smokers.

**Table 2:** Medical and family history of varicose veins among the participants

Medical and family history	No	%
Family history of varicose vein		
Yes	140	35.1%
No	259	64.9%
Diagnosed with varicose vein		
Yes	74	18.5%
No	325	81.5%
Smoking		
Yes	93	23.3%
No	306	76.7%

The most reported symptoms of varicose veins among the participants include pain in the legs, which increases especially during work (43.1%), Leg cramps at night (28.1%), feeling of heaviness in the legs (27.1%), feeling of numbness in the legs (24.1%), heat or itching in the legs (14.5%), leg pain that only goes away by taking painkillers (11.8%), and Itching around the veins in the legs (6.3%). A total of 38.6% of teachers had none of the varicose vein symptoms at all (Table 3).

**Table 3:** Reported symptoms of varicose veins among the participants

Symptoms	No	%
None of these	154	38.6%
Pain in the legs increases especially during work	172	43.1%
Leg cramps at night	112	28.1%
A feeling of heaviness in the legs	108	27.1%
A feeling of numbness in the legs	96	24.1%
Heat or itching in the legs	58	14.5%
Leg pain that only goes away by taking painkiller	47	11.8%
Itching around the vein in the legs	25	6.3%

**Figure 1:**
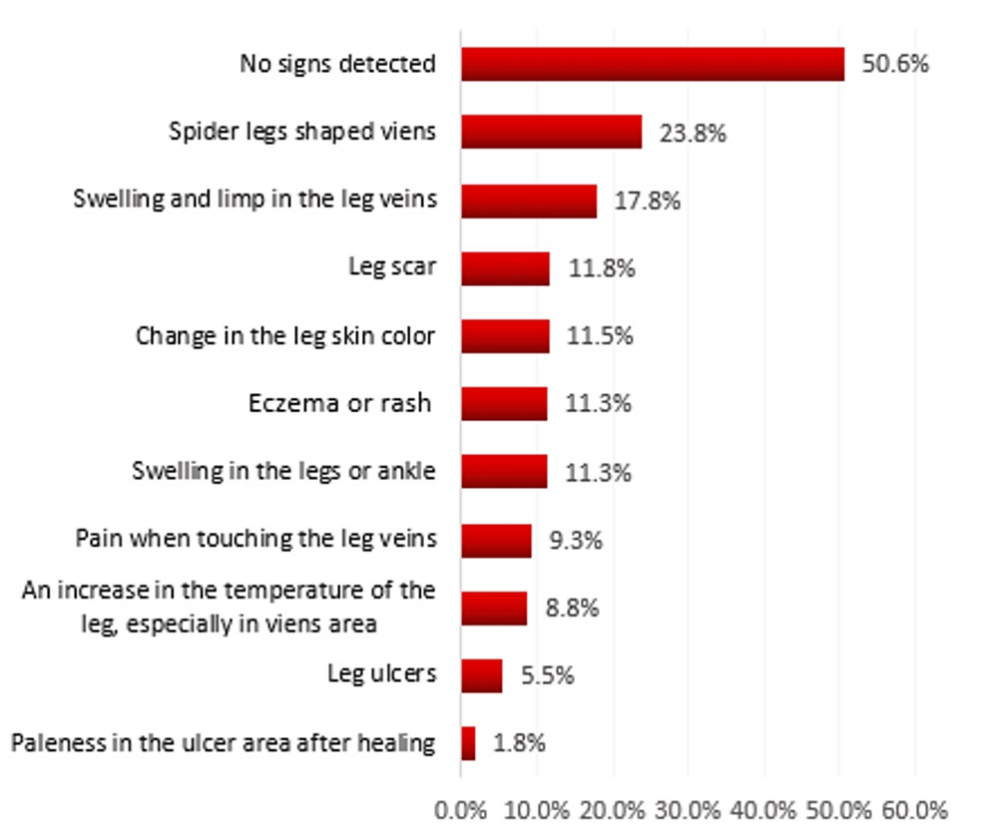
Signs of varicose veins by clinical examination among the participants

However, Figure 1 shows varicose vein signs detected on clinical examination of teachers, Al-Ahsa, Saudi Arabia. The most found signs were spider legs-shaped veins (23.8%), followed by swelling and limp in the leg veins (17.8%), leg scar (11.8%), change in the leg skin color (11.5%), eczema or rash on the legs (11.3%), swelling in the legs or ankle (11.3%), and pain when touching the leg veins (9.3%) while 50.6% had no signs.

The distribution of varicose vein signs and symptoms by teaching year is presented in Table 4. The most reported symptoms among teachers for four to seven years were leg scars (17.6%) and spider legs-shaped veins (17.6%) while the most among teachers for more than 16 years were spider legs-shaped veins (23.6%) and swelling and limp in the leg veins (19.5%) with no statistical significance (P=.598). As for signs, the most reported for teachers having taught for four to seven years were pain in the legs increases especially during work (38.2%) and feeling of heaviness in the legs (32.4%). Pain in the legs increases especially during work was the most reported symptom (50.6%) for those who taught for more than 16 years (P=.445).

**Table 4:** Distribution of the signs and symptoms of varicose veins by teaching year

Clinical findings	Teaching years								p-value
	4-7		8-11		12-15		16+		
	No	%	No	%	No	%	No	%	
Clinical examination signs									.598^$^
No signs detected	20	58.8%	46	47.4%	54	57.4%	82	47.1%	
Swelling and limp in the leg veins	2	5.9%	18	18.6%	17	18.1%	34	19.5%	
An increase in the temperature of the leg, especially in the veins area	1	2.9%	5	5.2%	9	9.6%	20	11.5%	
Leg scar	6	17.6%	10	10.3%	9	9.6%	22	12.6%	
Change in the leg skin colour	3	8.8%	12	12.4%	7	7.4%	24	13.8%	
Spider legs shaped veins	6	17.6%	26	26.8%	22	23.4%	41	23.6%	
Pain when touching the leg veins	1	2.9%	5	5.2%	9	9.6%	22	12.6%	
Swelling in the legs or ankle	4	11.8%	13	13.4%	8	8.5%	20	11.5%	
Leg ulcers	2	5.9%	6	6.2%	6	6.4%	8	4.6%	
Paleness in the ulcer area after healing	0	0.0%	0	0.0%	2	2.1%	5	2.9%	
Eczema or rash on the legs	3	8.8%	13	13.4%	9	9.6%	20	11.5%	
Symptoms teachers had									.445
Pain in the legs increases especially during work	13	38.2%	35	36.1%	36	38.3%	88	50.6%	
Leg cramps at night	9	26.5%	22	22.7%	24	25.5%	57	32.8%	
A feeling of heaviness in the legs	11	32.4%	22	22.7%	26	27.7%	49	28.2%	
Leg pain that only goes away by taking painkillers	3	8.8%	9	9.3%	14	14.9%	21	12.1%	
A feeling of numbness in the legs	7	20.6%	26	26.8%	25	26.6%	38	21.8%	
Heat or itching in the legs	3	8.8%	14	14.4%	10	10.6%	31	17.8%	
Itching around the veins in the legs	1	2.9%	7	7.2%	6	6.4%	11	6.3%	
None of these	15	44.1%	42	43.3%	40	42.6%	57	32.8%	

Table 5 measures the distribution of the signs and symptoms of varicose veins by teaching standing hours. Spider legs-shaped veins (24.1%) and swelling and limp in the leg veins (16.2%) were the most reported symptoms among teachers who stood for six hours or less compared to 22.9% and 22% of those who stood for more than six hours daily with recorded statistical significance (P=.048).

**Table 5:** Distribution of the signs and symptoms of varicose veins by teaching standing hours

Clinical findings	Standing hours / day				p-value
	< 6 hours		> 7 hours		
	No	%	No	%	
Clinical examination signs					.048*
No signs detected	149	51.4%	53	48.6%	
Swelling and limp in the leg veins	47	16.2%	24	22.0%	
An increase in the temperature of the leg, especially in veins area	24	8.3%	11	10.1%	
Leg scar	32	11.0%	15	13.8%	
Change in the leg skin color	32	11.0%	14	12.8%	
Spider legs-shaped veins	70	24.1%	25	22.9%	
Pain when touching the leg veins	23	7.9%	14	12.8%	
Swelling in the legs or ankle	26	9.0%	19	17.4%	
Leg ulcers	17	5.9%	5	4.6%	
Paleness in the ulcer area after healing	2	.7%	5	4.6%	
Eczema or rash on the legs	35	12.1%	10	9.2%	
Symptoms teachers had					.032*
Pain in the legs increases especially during work	112	38.6%	60	55.0%	
Leg cramps at night	79	27.2%	33	30.3%	
A feeling of heaviness in the legs	77	26.6%	31	28.4%	
Leg pain that only goes away by taking painkillers	29	10.0%	18	16.5%	
A feeling of numbness in the legs	67	23.1%	29	26.6%	
Heat or itching in the legs	43	14.8%	15	13.8%	
Itching around the veins in the legs	17	5.9%	8	7.3%	
None of these	120	41.4%	34	31.2%	

Last, Table 6 represents the factors associated with varicose veins among teachers in Al-Ahsa. Varicose veins were diagnosed among 23% of female teachers versus 14.8% of males (P=.037). Also, 24.5% of teachers who taught for 12-15 years were diagnosed with varicose veins compared to 8.8% of those who taught for four to seven years (P=.048). A total of 31.4% of teachers with a family history of varicose veins had varicose veins in comparison to 11.6% of others without (P=.001).

**Table 6:** Factors associated with varicose veins among the participants

Factors	Diagnosed with varicose veins				p-value
	Yes		No		
	No	%	No	%	
Age in years					.153
20-29	0	0.0%	11	100.0%	
30-39	15	14.9%	86	85.1%	
40-49	41	19.2%	173	80.8%	
50+	18	24.7%	55	75.3%	
Gender					.037*
Male	32	14.8%	184	85.2%	
Female	42	23.0%	141	77.0%	
Teaching years					.152
4-7	3	8.8%	31	91.2%	
8-11	20	20.6%	77	79.4%	
12-15	23	24.5%	71	75.5%	
16+	28	16.1%	146	83.9%	
Standing hours / day					.421
< 6 hours	51	17.6%	239	82.4%	
> 7 hours	23	21.1%	86	78.9%	
Body mass index					.211
Normal	10	11.9%	74	88.1%	
Overweight	42	20.4%	164	79.6%	
Obese	22	20.2%	87	79.8%	
Family history of varicose vein					.001*
Yes	44	31.4%	96	68.6%	
No	30	11.6%	229	88.4%	
Smoking					.940
Yes	17	18.3%	76	81.7%	
No	57	18.6%	249	81.4%	

## Discussion

Varicose veins are considered one of the most common manifestations of vascular disorders. The incidence of the disease is increasing, with multiple factors like age, sex, and prolonged standing [15]. Accordingly, the study aims to assess the risk of varicose veins among teachers in different schools in Al-Ahsa. This aim was addressed through a cross-sectional study with questionnaires. Our study shows that 53.6% of the participants belonged to the age group of 40 to 49, a relatively older age. Regarding gender, 54.1% of the participants were male and 45.9% were female. The majority of the participants taught for more than 16 years. More than three-fourths had a high BMI. Varicose veins were previously diagnosed in 18.5% of the participants. Likewise, a study conducted in Riyadh on the prevalence of varicose veins among nurses shows that 11% were previously diagnosed with the disease [16]. However, a higher prevalence is seen among teachers in Abha [12]. This difference might be because of the lifestyle differences between each city. In terms of symptoms, 43.1% of the teacher had leg pain during work. Similarly, 66% of nurses in different hospitals in Lebanon had leg pain during work [17]. Hence, pain is the most common presentation of varicose veins [18]. In addition, nocturnal cramps represented 28.1% of the participants. Likewise, in traffic police in Nepal, nocturnal cramp is about 32% [6]. The least presenting sign is itching. In terms of signs, the most presenting one is spider legs-shaped veins on the leg, which represent 23.8%. Similarly, 26% of city police in Belagavi have spider-shaped veins [19]. Furthermore, 17.8% of participants had swelling in the leg and pain. Surprisingly, dilated veins were the most common sign in traffic police of Nepal, representing 5.5%. Moreover, the third most common symptoms are leg scar (11.8%) followed by a change in skin color (11.5%) [6].

In our study, 11 teachers were between the ages of 20 and 29; none of them had been diagnosed with VV previously. A total number of 73 teachers aged 50+ years participated in this study; 18 of them were diagnosed with VV (24.7%). The percentage of teachers that were diagnosed with VV was less for those aged 40-49 (19.2%). However, the total number of teachers that participated in our study aged 40-49 was the highest (214 teachers). Old age is considered a risk factor for developing venous disease. As age increases, the prevalence of venous disease gets higher. This is due to the weakening of the calf muscles, which leads to increased pressure on superficial veins accompanied by the gradual deterioration of vessel walls over time [1]. In the United States, a study conducted to assess the prevalence of venous disease showed that the prevalence of VV among individuals younger than 30 years was less than 1% for men and less than 10% for women [20].

In the current study, there was a significant difference between male and female teachers regarding the diagnosis of VV. The percentage of diagnosed male teachers was 14.8%, whereas it was 23% for female teachers. Female gender was considered a risk factor for VV in the Kunie et al. study [21]. Similarly, several previous studies revealed that females are more prone to develop VV than males [20,22-24]. One of the major factors that contribute to the higher incidence of VV in women is pregnancy [25]. A higher incidence of VV is shown in parous women in comparison to nulliparous women [1].

Most of the teachers that participated in our study had taught for more than 16 years (total number of 174). Our results showed that 16.1% of those teachers were diagnosed with VV. Teachers that had taught for four to seven years were 34 in number, 8.8% were diagnosed with VV, which is the lowest percentage. However, the diagnosis of VV in relation to teaching years was insignificant in our study. Regarding standing hours per day, our result showed that 21.1% of the teachers who stood for more than seven hours per day were diagnosed with VV while the percentage was 17.6% for those who stood for less than seven hours. The standing position has been reported as an aggravating factor for VV in the European population in a study done by Krijnen et al. [26]. In Iran, a study among nurses reported four times increased odds of having VV for those who stood for more than four hours as compared to those who stood for less time [27].

Our study showed that 31.4% of teachers who have a family history of VV were diagnosed with the same condition, which is significantly different from those who don’t have a family history (11.6%). Even though specific genes haven't been identified yet, several studies demonstrated family history as an important risk factor for venous diseases [23,28-31]. Our result didn't show a statistically significant difference in teachers who smoked or those who had a high body mass index (BMI) in relation to the diagnosis of VV. This result is inconsistent with a study that involved 1806 patients with lower limb venous insufficiency and showed that smoking more than 10 cigarettes per day was associated with a higher prevalence of venous insufficiency [32]. Furthermore, previous studies identified obesity as a risk factor for VV [20,22].

This study didn’t include every school in Al-Ahsa, so it might not be representative of the prevalence of VV among all teachers in Al-Ahsa. Another limitation is that the measurement of hours spent in standing, height, and weight was self-reported. This could lead to an overestimation or underestimation by the participants. Lastly, participants didn’t undergo a Doppler ultrasound during the interview, which is an important objective measure to determine their vascular condition.

## Conclusions

Varicose veins is a common condition that has a high prevalence in females more than in males. Furthermore, teachers spend a lot of hours standing, which makes them vulnerable to developing this condition. As a result, further actions need to be taken in order to increase the level of education among teachers through mass media, campaigns, and further research studies to decrease its incidence and complications.
